# From Databases to Modelling of Functional Pathways

**DOI:** 10.1002/cfg.375

**Published:** 2004-03

**Authors:** Sergio Nasi

**Affiliations:** Istituto di Biologia e Patologia Molecolari CNR Università La Sapienza P. le A. Moro 5 Roma 00185 Italy

## Abstract

This short review comments on current informatics resources and methodologies in the study of functional pathways in cell biology. It highlights recent achievements in unveiling the structural design of protein and gene networks and discusses current approaches to model and simulate the dynamics of regulatory pathways in the cell.


**Comparative and Functional Genomics**
*Comp Funct Genom* 2004; **5**: 179–183.
Published online in Wiley InterScience (www.interscience.wiley.com). **DOI:** 10.1002/cfg.375

**Conference Review**

### From databases to modelling of functional pathways

Sergio Nasi*
*Istituto di Biologia e Patologia Molecolari CNR, Università La Sapienza, P. le A. Moro 5, 00185 Roma, Italy*

**Correspondence to: Sergio Nasi, Istituto di Biologia e Patologia Molecolari CNR, Università La Sapienza, P. le A. Moro 5, 00185 Roma, Italy. E-mail: sergio.nasi@uniroma1.it*

Received: 14 November 2003
Accepted: 24 November 2003

### Abstract

**This short review comments on current informatics resources and methodologies in the study of functional pathways in cell biology. It highlights recent achievements in unveiling the structural design of protein and gene networks and discusses current approaches to model and simulate the dynamics of regulatory pathways in the cell. Copyright © 2004 John Wiley & Sons, Ltd.**

**Keywords:** **cell biology; bioinformatics; databases; gene networks; protein interactions; functional pathways; modelling**

Understanding how genes interact to perform specific biological processes is a major challenge in biology. It is felt that this is becoming possible due to the large amount of information generated by genomic sequencing, protein interaction and gene expression studies, and stored in public databases (**www.ncbi.nlm.nih.gov/GenBank**; **www.ncbi.nlm.nih.gov/LocusLink**; **www.ncbi.nlm.nih.gov/UniGene**; **http://us.expasy.org/sprot**; **www.ensembl.org**; **www.ebi.ac.uk**; **www.yeastgenome.org/**; **www.arabidopsis.org/**; **www.wormbase.org/**; **http://flybase.bio.indiana.edu/**; **www.informatics.jax.org**; **http://rgd.mcw.edu/**; **http://genome-www5.stanford.edu/MicroArray/SMD/**). Achieving this objective will require data to be organized in a more understandable structure. Data representation in the form of networks or functional pathways, and modelling their dynamic behaviour, is expected to give a better insight into the complex patterns of gene–protein interactions. At the same time, such models are expected to revolutionize drug screening, and the identification of functional pathways involved in pathogenesis will facilitate the rational design of therapies [11].

### Databases

The effort of creating biological pathway databases and providing informatics tools for their analysis has been undertaken by public and private initiatives, such as Transpath (**www.biobase.de**), Biocarta (**www.biocarta.com**), GenMAPP (**www.genmapp.org**), aMaze (**www.amaze.ulb.ac.be**) and the Alliance for Cellular Signaling (AfCS:**www.afcs.org**). The AfCS consortium, which is presently focused on lymphocyte and cardiac myocyte signalling, has the overall goal to understand the relationships between sets of inputs and outputs that vary both temporally and spatially. This will involve identification of all the proteins that comprise the various signalling systems, the assessment of information flow in both normal and pathological states, and the reduction of the data into a set of theoretical models. The aMaze project of an omni-comprehensive, object-orientated data model is implemented in both MySQL and Oracle languages. It aims at representing functional and physical interactions among biochemical entities mapped onto their cellular and tissue locations. It also attempts to provide a workbench for analysing networks of cellular processes, such as metabolic pathways, protein–protein interactions, gene regulation, transport and signal transduction. Most of the pathway data presently stored in the database relate to yeast and bacterial cells. A complication in pathway analysis results from network component compartmentalization in space and time, both at the cellular level

Copyright © 2004 John Wiley & Sons, Ltd.

and between the cells of a multicellular organism. This aspect is taken into account by the MGEIR (Mouse Gene Expression Information Resource) Project, developed collaboratively by the mouse Gene eXpression Database at the Jackson Laboratory and the Edinburgh Mouse Atlas Project [5] (**www.informatics.jax.org**; **http://genex.hgu.mrc.ac.uk**). Its goal is to illustrate molecular networks in the context of the whole organism, by providing a unified resource to store, display and analyse mouse developmental gene expression information. Data is accessible both as text, using standardized names for anatomical terms, and as original published images of *in situ* hybridization, RT–PCR, immuno-histochemistry, etc.

### Gene and protein network architecture

Gene or protein networks are more easily understood when represented as graphs, in which nodes are genes or proteins, and arcs (edges) are relationships between nodes. Depending on the case, edges can have direction and weight. Data from high-throughput protein interaction screens and DNA microarray experiments, as well as tools for mining information in the scientific literature, have supported the elucidation of the structural design of networks, an important step towards modelling and understanding cellular control systems. By employing controlled vocabularies (**www.geneontology.org**) linked to gene symbols, it is possible to mine qualitative information: automatic query methods have been used to extract and structure knowledge from publicly available gene/protein and text databases. This allows the creation of a co-citation network [17], under the assumption that co-occurrence of the names of biological entities, such as genes and proteins, in the same Medline abstract may reflect biologically meaningful relationships, thus unveiling hidden patterns. Databases and accompanying web tools for mining relationships in the scientific literature are provided by the PubGene and BiblioSphere sites (**www.pubgene.org**; **www.genomatix.de**). Functional assignment of proteins can be assisted by literature data mining, and by informatics approaches such as automatic annotation (**www.pdg.cnb.uam.es/blaschke/cgi-bin/abx**) or *in silico* two-hybrid, which takes into account the co-evolution of sequence features (**www.pdg.cnb.uam.es/i2h**).

Due to their importance in cell physiology, considerable efforts are being devoted to large-scale mapping of protein interaction networks by yeast two hybrid screens [29] (**www.hybrigenics.com/**; **http://portal.curagen.com/cgi-bin/interaction/flyHome.pl**), by purification of protein complexes followed by mass spectrometry [10,13] (**www.cellzome.com**) and, hopefully, using protein chips (**http://bioinfo.mbb.yale.edu/proteinchip**). Whereas representations of the network of protein complexes from large scale pull-down experiments is thought to be more accurate than representation of binary interactions from two-hybrid screens, both fail to correctly reproduce all the interactions described in the literature, so their utility in pathway design is limited. As a matter of fact, the resulting protein interaction maps were shown to be incomplete and contradictory to a significant extent, containing a large amount of spurious interactions and missing a large number of true interactions, varying from 15% to 85% according to the dataset [9]. Similar problems are likely to affect protein interaction databases such as BIND, DIP and MINT, which also contain hand-checked information gathered from the scientific literature (**http://dip.doe-mbi.ucla.edu/**; **www.blueprint.org/bind/bind.php**; **http://cbm.bio.uniroma2.it/mint/**). The validity of the interaction data can be improved with the use of structural information about protein complexes, available at MIPS (**http://mips.gsf.de/**). Different representations of protein networks tend to have a small overlap, estimated to be around 20%. Although it appears that no-one at present is able to manage the complexity of protein interaction maps, platforms are being designed to help simplify their analysis (**www.hybrigenics.com/**). It will be valuable to have databases that take into account the uncertainty of the current data in both literature and genome-wide experiments, by describing the networks in some sort of probabilistic terms. In this context, a Bayesian networks approach was employed for predicting a protein interaction network from a number of genomic features [16] (**http://bioinfo.mbb.yale.edu/genome/intint/**). Since existing datasets are so inaccurate, the intersection of different datasets and integration of information from a variety if sources is utilized to improve the accuracy and increase coverage of interactions. To facilitate the work of combining and verifying data from different sources,

Copyright © 2004 John Wiley & Sons, Ltd.

the Proteomics Standards Initiative (PSI) aims to define community standards for data representation (**http://psidev.sourceforge.net**).

The structure of gene interaction networks is not measured directly, but must be reconstructed by reverse engineering. The primary data source has been microarray analysis of gene expression profiles, a technique yielding a wealth of information, but somehow noisy and often incomplete (**http://genome-www5.stanford.edu/MicroArray/SMD/**; **www.ebi.ac.uk/arrayexpress/**; **www.hgmp.mrc.ac.uk/Research/Microarray/index.jsp**). Microarray data analysis presents the challenge of revealing functional patterns in the chaos that is gene expression. The starting point is a gene expression data matrix, utilized by clustering algorithms to identify co-expressed genes, which are thought to be regulated by shared transcription factors (**http://genexpress.stanford.edu**). Although powerful for organizing data, such algorithms, by themselves, are unfit for model building since they do not relate gene expression values to a given functional state. Graph theory, supervised learning and other statistical and computational approaches have been adopted to make predictions and to reconstruct gene regulation networks from microarray data [14,26]. The uncertainty inherent in these data is taken into account by computational tools such as rough sets (**http://rosetta.lcb.uu.se**), which are used in supervised learning to build if–then rules. Such rules are then used to model the relationship between time course of gene expression and involvement of a gene in a given biological process ([15] **www.lcb.uu.se/~hvidsten/bioinf_cho**). A graph theory-based approach was used to reconstruct a gene network from microarray data of single deletion mutants in yeast ([24] **http://industry.ebi.ac.uk/~schlitt/draft/title.html**). Precious hints on the function of genes can be derived from gene co-expression, when applied in an evolutionary context. Starting from metagenes (sets of best orthologues identified by BLAST searches), probabilistic methods were used to construct a gene co-expression network, subnets of which may be associated with particular biological pathways ([27] **http://cmgm.stanford.edu/~kimlab/multiple-species**).

Although it might be possible in principle, network reconstruction based solely on microarray experiments proved very hard to achieve, pointing to the utility of incorporating information on transcription factor binding to gene promoters ([31] **www.math.uah.edu/stat**). Interaction between transcription factors and their DNA binding sites may be deduced from computational analysis of binding sites in promoter sequences [22], with the assistance of transcription factor databases such as Transfac (**www.biobase.de**) and tools such as MathInspector and ElDorado (**www.genomatix.de**). Direct mapping of these interactions is also possible, through genome-wide chromatin immunoprecipitation (ChIP–chip technology). This technique has revealed recurrent regulatory motifs that serve as the building blocks of complex gene networks [19]. Analysis of the genomic distribution of transcripts and of factor binding sites can reach an extremely high resolution by means of genomic tiling arrays, oligonucleotide arrays containing probes spaced on average every 5 bp along the genome. The application of these techniques to mammalian genomes has revealed many transcripts arising from templates outside of known and predicted genes, as well as anti-sense RNA transcripts; it has also been observed that DNA fragments that are cross-linked to a given transcription factor frequently do not have a recognizable binding site (TM Gingeras and SM Weissman, personal communications).

Methods have been devised to extract regulatory information from binding data and to find synergistic motif combinations in the promoters of co-regulated genes ([19,22,23] **http://web.wi.mit.edu/young/regulator_network**). More advanced methods, such as the genetic regulatory modules (GRAM [2]) and the module networks algorithms ([25] **http://robotics.stanford.edu/~erans/module_nets**) incorporate both DNA-binding and gene expression data, allowing the selection of sets of genes that share a common group of transcription factors and also have similar expression profiles.

Both protein and gene interaction networks appear to be scale-free, the connectivity of their nodes following a power law; therefore, they have small world properties like many other networks found in nature. Such global views, although fascinating, do not always appear of immediate utility for biologists, since they give only a general impression of the network operation and lack crucial details [3].

Copyright © 2004 John Wiley & Sons, Ltd. 

### Modelling of cellular pathways

Depicting sets of molecular interactions as static graphs does not reveal the dynamics of events within cells. The myriad of data now available has stimulated attempts to design a computer replica of a living cell, by including everything that is known in one description of an entire cell biological network. Several projects aim to develop theoretical supports, technologies and software platforms for whole cell simulation. The Simulation Environment of the open source E-Cell Project (**www.e-cell.org**) can integrate different simulation algorithms, including differential equation-based models, diffusion-reaction and cellular automata. Another framework for modelling and simulation of cell biological models, the SmartCell, is under development in the Serrano lab at EMBL (**www.embl-heidelberg.de/emblGroup/researchReport/rr02_54.pdf**). It is expected to provide a suitable model description format. The virtual cell (**www.nrcam.uchc.edu/**) provides a Java-based modelling and simulation environment, in which users can create biological models of various types and run simulations on a remote server; another tool allows users to translate the initial biological description into a set of concise mathematical problems. The silicon cell consortium (**www.siliconcell.net**) has embraced the philosophy of always starting from real molecular data and of computing the implications for systems biology. The idea that this can be done simply by computing what is known appears over-optimistic, since the number of molecular processes to be considered is too high, and this approach suffers from the inability to take into account interactions not yet discovered. Clearly this must be integrated by top-down model construction, which also represents a way of integrating data in a more understandable structure [28]. An intuitive understanding of genetic regulatory networks, which involve many components connected through interlocking loops, is hard to obtain. Computational methods like GRAM [2] and module networks [25] can give a first hint on the dynamic behaviour of gene expression regulatory networks and may represent a tool for the development of more advanced dynamic models. Formalisms that have been employed to model these networks include directed graphs, Bayesian and Boolean networks, rule-based formalism and various kinds of differential equations ([8] **www.berkeleymadonna.com**). Each one has its advantages and drawbacks that reflect the difficulty of incorporating the different features of gene regulatory networks, with aspects that appear to comply to a Boolean logic (on–off switches at discrete time steps) and others that are better described by differential equation models. Mixed feature models, such as the finite state linear model [4], and simplified, qualitative methods, such as the genetic network analyser [7], are an attempt to get closer to the reality of gene networks, whose behaviour has been faithfully reproduced in the case of the λ-phage lysis/lysogeny switch [4] and the initiation of sporulation in *Bacillus subtilis* [7].

In engineering applications, the challenge of understanding the behaviour of a complex network is facilitated by analysing them within a modular framework. The network is divided into subsets of nodes that have strong interactions and a common function, named 'modules' or 'functional units'. Fortunately, modularity appears to be a characteristic of several biological networks, together with other structural principles that may facilitate analysis, such as robustness and use of recurring circuit elements [1,12,21]. A modular strategy was applied to model simple gene networks and EGF signalling [18]. In this last case, the mitogen-activated protein kinase (MAPK) cascade was divided into three modules that interact through communication intermediates; the module–module connection strengths can be obtained by measuring global responses to specific perturbations of the cascade. Robustness and modularity were keys to the successful modelling of the segmentation polarity network, which involves interactions among the products of five genes [30]. To formulate a dynamic model in that case required 136 equations with 50 free parameters that were in large part unknown, as is frequently the case, and which might have spanned several orders of magnitude. Describing the fission yeast cell cycle wiring diagram involved a dozen differential equations, with about 30 kinetic parameters [28]. A combination of experimental and computational methods have made it possible to unveil the operation of other biological pathways, such as those that regulate *Saccharomyces cerevisiae* [19] and *Caulobacter crescentus* [20] cell cycle, or that dictate endoderm development in sea urchin embryo through a Byzantine control system of over 40 genes [6].

Copyright © 2004 John Wiley & Sons, Ltd. 

Going from these examples to more complex pathways or, in the long run, to *in silico* whole cell models, will depend on an increasing availability of better structured databases and on finding out innovative mathematics solutions to modelling and simulation.

### Acknowledgements

This work was partially supported by FIRB and FIRS grants from the Italian Ministry of University and Research (MIUR), and by a grant from the Italian Association for Cancer Research (AIRC).

### References

1. Alon U. 2003. Biological networks: the tinkerer as an engineer. *Science* **301**: 1866–1867.
2. Bar-Joseph Z, Gerber GK, Lee TI, *et al*. 2003. Computational discovery of gene modules and regulatory networks. *Nature Biotechnol* **21**: 1337–1342.
3. Bray D. 2003. Molecular networks: the top-down view. *Science* **301**: 1864–1865.
4. Brazma A, Schlitt T. 2003. Reverse engineering of gene regulatory networks: a finite state linear model. *Genome Biol* **4**(6): P5 (**http://genomebiology.com/2003/4/6/P5**).
5. Davidson D, Baldock R. 2001. Bioinformatics beyond sequence: mapping gene function in the embryo. *Nature Rev Genet* **2**: 409–417.
6. Davidson EH, Rast JP, Oliveri P, *et al*. 2002. A genomic regulatory network for development. *Science* **295**: 1669–1678.
7. De Jong H, Geiselmann J, Hernandez C, Page M. 2003. Genetic Network Analyzer: qualitative simulation of genetic regulatory networks. *Bioinformatics* **19**(3): 336–344.
8. De Jong H. 2002. Modeling and simulation of genetic regulatory systems: a literature review. *J Comput Biol* **9**(1): 67–103.
9. Edwards AM, Kus B, Jansen R, *et al*. 2002. Bridging structural biology and genomics: assessing protein interaction data with known complexes. *Trends Genet* **18**(10): 529–536.
10. Gavin A, Superti-Furga G. 2003. Protein complexes and proteome organization from yeast to man. *Curr Opin Chem Biol* **7**: 21–27.
11. Hahn W, Weinberg R. 2002. Modelling the molecular circuitry of cancer. *Nature Rev Cancer* **2**: 331–341.
12. Hartwell L, Hopfield J, Leibler S, Murray A. 1999. From molecular to modular cell biology. *Nature* **402**: C47–C52.
13. Ho Y, Gruhler A, Heilbut A, *et al*. 2002. Systematic identification of protein complexes in *Saccharomyces cerevisiae* by mass spectrometry. *Nature* **415**: 180–183.
14. Huang E, Ishida S, Pittman J, *et al*. 2003. Gene expression phenotypic models that predict the activity of oncogenic pathways. *Nature Genet* **34**: 226–230.
15. Hvidsten T, Lægreid A, Komorowski J. 2003. Learning rule-based models of biological process from gene expression time profiles using Gene Ontology. *Bioinformatics* **19**(9): 1116–1123.
16. Jansen R, Yu H, Greenbaum D, *et al*. 2003. A Bayesan networks approach for predicting protein–protein interactions from genomic data. *Science* **302**: 449–453.
17. Jenssen T, Laegreid A, Komorowski J, Hovig E. 2001. A literature network of human genes for high-throughput analysis of gene expression. *Nature Genet* **28**: 21–28.
18. Kholodenko B, Kiyatkin A, Bruggeman F, *et al*. 2002. Untangling the wires: a strategy to trace functional interactions in signaling and gene networks. *Proc Natl Acad Sci USA* **99**(20): 12 841–12 846.
19. Lee T, Rinaldi N, Robert F, *et al*. 2002. Transcriptional regulatory networks in *Saccharomyces cerevisiae*. *Science* **298**: 799–804.
20. McAdams H, Shapiro L. 2003. A bacterial cell-cycle regulatory network operating in time and space. *Science* **301**: 1874–1877.
21. Milo R, Shen-Orr S, Itzkovic S, *et al*. 2002. Network motifs: simple building blocks of complex networks. *Science* **298**: 824–827.
22. Pilpel Y, Sudarsanam P, Church GM. 2001. Identifying regulatory networks by combinatorial analysis of promoter elements. *Nature Genet* **29**: 153–159.
23. Qian J, Lin J, Luscombe NM, Yu H, Gerstein M. 2003. Prediction of regulatory networks: genome-wide identification of transcription factor targets from gene expression data. *Bioinformatics* **19**(15): 1917–1926.
24. Rung J, Schlitt T, Brama A, Freivalds K, Vilo J. 2002. Building and analysing genome-wide gene disruption networks. *Bioinformatics* **18**(2): S202–S210.
25. Segal E, Shapira M, Regev A, *et al*. 2003. Module networks: identifying regulatory modules and their condition-specific regulators from gene expression data. *Nature Genet* **34**: 166–176.
26. Soinov L, Krestyaninova M, Brazma A. 2003. Towards reconstruction of gene networks from expression data by supervised learning. *Genome Biol* **4**: R6.
27. Stuart M, Segal E, Koller D, Kim S. 2003. A gene co-expression network for global discovery of conserved genetic modules. *Science* **302**: 249–255.
28. Tyson J, Chen K, Novak B. 2003. Sniffers, buzzers, toggles and blinkers: dynamics of regulatory and signaling pathways in the cell. *Curr Opin Cell Biol* **15**(2): 221–231.
29. Uetz P, Giot L, Cagney G, *et al*. 2000. A comprehensive analysis of protein–protein interactions in *Saccharomyces cerevisiae*. *Nature* **403**: 623–627.
30. von Dassow G, Meir E, Munro EM, Odell GM. 2000. The segment polarity network is a robust developmental module. *Nature* **406**: 188–192.
31. Wyrick J, Young R. 2002. Deciphering gene expression regulatory networks. *Curr Opin Genet Dev* **12**: 130–136.

Copyright © 2004 John Wiley & Sons, Ltd. 
